# Predicting Survival in Repaired Tetralogy of Fallot: A Lesion-Specific and Personalized Approach

**DOI:** 10.1016/j.jcmg.2021.07.026

**Published:** 2022-02

**Authors:** Sarah Ghonim, Michael A. Gatzoulis, Sabine Ernst, Wei Li, James C. Moon, Gillian C. Smith, Ee Ling Heng, Jennifer Keegan, Siew Yen Ho, Karen P. McCarthy, Darryl F. Shore, Anselm Uebing, Aleksander Kempny, Francisco Alpendurada, Gerhard P. Diller, Konstantinos Dimopoulos, Dudley J. Pennell, Sonya V. Babu-Narayan

**Affiliations:** a Royal Brompton and Harefield Hospitals, Guy’s and St. Thomas’ NHS Foundation Trust London, United Kingdom; b National Heart Lung Institute, Imperial College London, United Kingdom

**Keywords:** CMR, late gadolinium enhancement, risk stratification, sudden cardiac death, tetralogy of Fallot, ventricular tachycardia, AUC, area under the curve, BNP, B-type natriuretic peptide, CMR, cardiovascular magnetic resonance, ECG, electrocardiogram, EF, ejection fraction, ICD, implantable cardiac defibrillator, LGE, late gadolinium enhancement, LV, left ventricle, NSVT, nonsustained ventricular tachycardia, PR, pulmonary regurgitation, rTOF, repaired tetralogy of Fallot, RV, right ventricle, RVOT, RV outflow tract, SCD, sudden cardiac death, VA, ventricular arrhythmia, VT, ventricular tachycardia

## Abstract

**Objectives:**

This study sought to identify patients with repaired tetralogy of Fallot (rTOF) at high risk of death and malignant ventricular arrhythmia (VA).

**Background:**

To date there is no robust risk stratification scheme to predict outcomes in adults with rTOF.

**Methods:**

Consecutive patients were prospectively recruited for late gadolinium enhancement (LGE) cardiovascular magnetic resonance (CMR) to define right and left ventricular (RV, LV) fibrosis in addition to proven risk markers.

**Results:**

The primary endpoint was all-cause mortality. Of the 550 patients (median age 32 years, 56% male), 27 died (mean follow-up 6.4 ± 5.8; total 3,512 years). Mortality was independently predicted by RVLGE extent, presence of LVLGE, RV ejection fraction ≤47%, LV ejection fraction ≤55%, B-type natriuretic peptide ≥127 ng/L, peak exercise oxygen uptake (V0_2_) ≤17 mL/kg/min, prior sustained atrial arrhythmia, and age ≥50 years. The weighted scores for each of the preceding independent predictors differentiated a high-risk subgroup of patients with a 4.4%, annual risk of mortality (area under the curve [AUC]: 0.87; *P* < 0.001). The secondary endpoint (VA), a composite of life-threatening sustained ventricular tachycardia/resuscitated ventricular fibrillation/sudden cardiac death occurred in 29. Weighted scores that included several predictors of mortality and RV outflow tract akinetic length ≥55 mm and RV systolic pressure ≥47 mm Hg identified high-risk patients with a 3.7% annual risk of VA (AUC: 0.79; *P* < 0.001) RVLGE was heavily weighted in both risk scores caused by its strong relative prognostic value.

**Conclusions:**

We present a score integrating multiple appropriately weighted risk factors to identify the subgroup of patients with rTOF who are at high annual risk of death who may benefit from targeted therapy.

Premature death, including sudden cardiac death (SCD) and ventricular arrhythmia (VA), are devastating late occurrences for the growing population of adults living with repaired tetralogy of Fallot (rTOF) (1, 2, 3). Despite decades of research, in clinical practice, risk stratification for survival and life-threatening ventricular tachycardia (VT) remains elusive, with deaths still occurring.

The lack of large prospective studies to support evidence-based decisions and therefore how to apply current clinical guidelines to individual patients is problematic (4,5). Pulmonary regurgitation (PR) is now a widely recognized hemodynamic substrate for VA and SCD, and considerable progress has been made in defining timing of pulmonary valve implantation (PVR) to counter it. Timely PVR alone, however, does not appear to abort the SCD risk, as myocardial fibrosis, a clear arrhythmic substrate for macro–re-entry VT remains (6,7). Multiple hemodynamic, structural, and electrophysiological risk factors have been described, although none sensitive and or specific enough to predict VT and SCD when used in isolation (8, 9, 10). The challenge, therefore, remains in selecting high-risk patients from a much larger rTOF cohort that overall has only a 0.15% annual risk of SCD (11) without contaminating the lives of remaining patients with implantable cardiac defibrillator (ICD) therapy with the physical and mental health issues associated with living with an ICD (11, 12, 13). A robust risk scheme integrating multiple risk factors appropriately is required (4).

Noninvasive assessment of VT substrates has been made possible using late gadolinium enhancement (LGE) cardiovascular magnetic resonance (CMR). We (14), and others, demonstrated association of LGE with right ventricular (RV) dysfunction, impaired exercise capacity, increased neurohormonal activation, and, importantly, sustained arrhythmia (atrial or ventricular) or syncope in cross-sectional studies (14). The aim of this prospective study was to examine the prognostic value of LGE and to construct a weighted-risk score for death and VA incorporating all independent risk factors in order to help identify high-risk patients who require consideration of ICD, and other interventions, such as preventive VT ablation or further optimization of heart failure therapy.

## Methods

### Patients

We recruited prospectively consecutive patients with rTOF ≥16 years of age between 2002 and 2019 for LGE CMR in addition to standard tertiary care (including 3 with dual-chamber permanent pacemaker [1 conditional, 2 conventional]). Patients with contraindication to cardiovascular magnetic resonance or gadolinium were excluded. Patients provided written informed consent. The study was approved by the local ethics committee and conducted in accordance with the Declaration of Helsinki.

### CMR image acquisition and analysis

**Figure 1:**
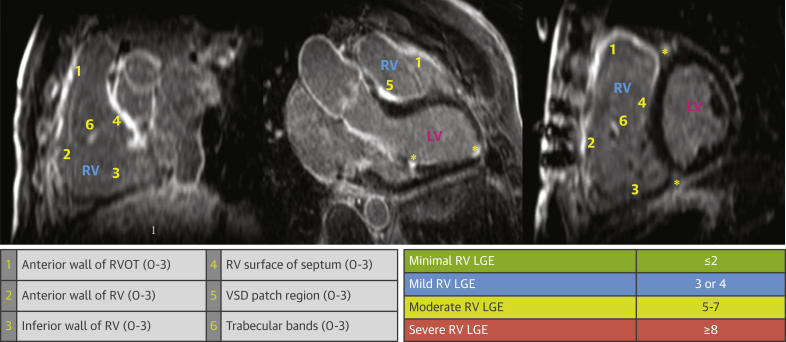
Segmental Scoring System for RV LGE Example of severe RV LGE extent in our study participant. The total RV LGE score was 12. RV-LV insertion point LGE, LV apical vent site LGE, and LV papillary muscle enhancement (**asterisks**) were not included in the score and were common. Patients with a total RV LGE score ≥8 were in the top quartile for RV LGE burden and were graded as severe extent. Patients with a total RV LGE score 5-7 were graded as moderate extent where score of 5 was the median. A total RV LGE score <5 was deemed as minimal or mild. The segmental system used for scoring RV LGE has been previously published. The RV is divided into 6 segments (**yellow numbers 1-6**). Regions of RV LGE were scored according to linear extent (0 = no enhancement, 1 = up to 2 cm, 2 = up to 3 cm, 3 = 3 or more cm in length) and number of trabeculations enhanced including the moderator band (0 = no enhancement, 1 = 1 trabeculation, 2 = 2-4). Scoring of LVLGE was performed using the universally accepted 17-segment LV model (14). Points were attributed to the proportion of LGE present in each myocardial segment, as visually judged: 0 = no LGE, 1 = up to 25%, 2 = up to 50%, 3 = up to 75%, and 4 = up to and including 100% of the myocardium enhanced. LGE = late gadolinium enhancement; LV = left ventricle; RV = right ventricle.

A standardized CMR protocol for rTOF assessment was acquired in all patients in line with our published protocol (14). Short-axis Cines were acquired for calculation of volumes with 7-mm slice thickness and 3-mm gap (spatial resolution 1.9 × 1.9 × 7 mm). Gadolinium-DTPA 0.1 mmol/kg intravenously was administered, and images were acquired from at least 8 to 10 minutes typically until at least 25 minutes after gadolinium was given. LGE images were obtained using an inversion-recovery gradient-echo sequence (spatial resolution 0.7 × 0.7 × 7 mm) with inversion times optimized to null normal myocardium by meticulous visual inspection of each image. Images were repeated in 2 separate phase-encoding directions or cross-cut to exclude possible areas of artifact and to define subtle RVLGE. Ventricular volumes analysis excluded trabeculations from RV and left ventricular (LV) blood pool (14). Maximum length of RV outflow tract (RVOT) akinetic region and indexed right atrial area (RAAi) were measured as previously reported (9). RVLGE was semiquantified by 2 experienced operators blinded to clinical data using the previously published segmental scoring system designed by our group to account for the unique geometry of the RV (Figure 1) (14). LGE was considered present when in locations that either did not alter when re-imaged in the same plane with a phase-swap or remained visible in a second orthogonal or cross-cut plane. LVLGE was scored using the standard 17-segment LV model. LVLGE related to apical vent at time of surgery and RV/LV septal insertion points were not included in the analysis, as previously described (14). Interstudy reproducibility of RV and LV LGE scoring was tested by repeating scans and analysis in 20 patients performed by 2 different operators blinded to previous study (14). The index LGE CMR study performed at the start of each patient’s recruitment was included for analysis.

### Standard of care investigations

As part of routine care, patients undergo periodic 12-lead electrocardiograms (ECG), blood sampling for B-type natriuretic peptide (BNP), echocardiography, and cardiopulmonary exercise testing (15, 16, 17). RV restrictive physiology was defined by laminar anterograde Doppler flow in the pulmonary artery in late diastole present throughout the respiratory cycle (“a” wave). We further classified restrictive RV physiology into the so-called primary phenotype and secondary phenotypes with a small or large RV, respectively. Tricuspid regurgitation, RV systolic pressure (RVSP), tricuspid annulus planar excursion, and LV diastolic filling parameters were reported from echocardiography. Peak oxygen uptake (peak VO_2_) was included when respiratory exchange ratio was >1. Ambulatory ECG Holter monitoring was performed if clinically indicated. Nonsustained VT (NSVT) was defined as ≥3 consecutive ventricular beats ≥100 beats per minute for ≤30 seconds’ duration and sustained atrial arrhythmia was defined as ≥30 seconds’ duration. These were recorded from Holter readings, routine pacemaker device interrogations, and medical notes.

### Follow-up and endpoints

Follow-up time was calculated from the time of index CMR until an endpoint had occurred or the last contact with the patient. Mortality status was verified from the United Kingdom Health and Social Care Information Service. Cause of death was established from death certificates, communication with the patient’s primary care physician, and review of medical records.

The primary endpoint was all-cause mortality. SCD was defined as an “unexpected death” in the absence of progressive cardiac deterioration. The secondary endpoint was ventricular arrhythmia (VA), which included SCD, resuscitated ventricular fibrillation (VF), and clinically sustained VT. Only the first event for each patient was included in the analysis. Clinically sustained VT was defined as VT ≥30 seconds’ duration or causing hemodynamic compromise requiring cardioversion. Resuscitated VF was defined as an appropriate shock for VF or successful resuscitation following VF cardiac arrest.

### Statistical analysis

Continuous data are summarized as mean ± SD and median (IQR). Comparison between groups was made by chi-square, Fisher exact test. or Mann-Whitney *U* test. A 2-sided *P <* 0.05 was considered statistically significant. Intraclass correlation coefficient was used to assess reproducibility of LGE scoring. The association between variables and outcome was tested using Cox proportional hazards model. Risk is a continuum throughout a patient’s life and we acknowledge inherent limitations of having a categorical risk score design; nevertheless, this was chosen as it is user-friendly to apply clinically. Significant univariable predictors of outcome were converted to categorical variables (based on highest/lowest quartile or decile). Relative beta coefficient values of only the variables that remained independently predictive of the outcomes and unrelated to one another in bivariable analysis were used as a guide to assign a relative weighting to each variable. A weighted-risk stratification score was thus derived for mortality and the secondary endpoint VA, respectively. Receiver operating characteristic curves were used to determine whether risk scores for mortality and VA could be used to predict outcome. The thresholds for each risk category for mortality and VA were selected based on sensitivity and specificity for outcome.

Cox proportional hazards survival plots where generated to illustrate the survival differences between high-, intermediate-, and low-risk categories for mortality and VA. Patients who already had the endpoints were censored at baseline. The relative performance of our risk score was compared using receiver-operating characteristic analysis with 3 existing risk scores (8,11,18) and our proposed score with and without LGE.

## Results

### Study population

A total of 550 patients with rTOF studied with LGE CMR (median age 32 years [23-42 years]; 57% male) were prospectively followed for a mean duration of 6.4 years (±5.8 years). This represents 3,512 patient-years of follow-up. Patient characteristics are summarized in Table 1. RV LGE was found at the surgical sites in all patients: 98% in the RVOT and 100% in the VSD patch site. LGE was found in RV trabeculations and moderator band in 176 (32%). Nonapical vent LV LGE was found in 7% (n = 41). Of these, infarct-related LGE was found in 8, papillary muscle/trabeculation LGE in 20, and LGE related to extension of VSD patch or spontaneously closed VSD. Interobserver reproducibility of LGE scoring was highly reproducible (intraclass correlation coefficient 0.97 and 1 for the RV and LV, respectively) (14). Clinical events at study end in relation to RVLGE extent are summarized in Supplemental Table 1.

**Table 1:** Patient Characteristics, Univariable Predictors of Mortality, and VA

	All Patients^a^ (N = 550)	All-Cause Mortality		VA	
		HR (95% CI)	*P* Value	HR (95% CI)	*P* Value
Age at scan, y	32 (23-42)	1.06 (1.03-1.09)	**<0.001**	1.04 (1.02-1.06)	**<0.001**
Age ≥50 y	66 (12)	4.9 (2.2-10.9)	**<0.001**	3.1 (1.3-7.4)	**0.01**
Male	312 (57)	1.40 (0.60-3.00)	0.30	0.46 (0.20-1.08)	0.07
Palliative shunt	196 (36)	2.3 (1.1-5.3)	**0.03**	1.6 (0.7-3.4)	0.20
Age of repair, y	4 (1.5-8)	1.06 (1.03-1.09)	**<0.001**	1.04 (1.004-1.07)	**0.03**
Age at repair ≥2 y	391 (72)	1.6 (0.5-4.6)	0.40	2.1 (0.7-5.9)	0.20
Ventriculotomy	418 (92)	22.7 (0.02-183.24)	0.40	1.1 (0.26-4.70)	0.90
Transannular patch	145 (39)	0.3 (0.10-1.05)	0.06	0.6 (0.30-0.50)	0.30
RVOT patch	125 (34)	1.4 (0.5-3.9)	0.50	1.6 (0.6-4.1)	0.30
RV-PA conduit	75 (21)	0.4 (0.5-4.4)	0.50	0.5 (0.1-2.2)	0.40
Redo surgery to implant pulmonary valve^b^	152 (27)	1.90 (0.80-4.80)	0.10	2.20 (1.04-4.80)	**0.04**
NYHA functional class ≥II	92 (17)	5.4 (2.5-11)	**<0.001**	3.9 (1.9-8.2)	**<0.001**
QRS duration,^c^ ms	153 (138-165)	1.01 (0.90-1.04)	0.40	1.01 (0.90-1.03)	0.30
QRS duration >180 ms	46 (8)	1.2 (0.4-3.6)	0.70	1.4 (0.5-4.0)	0.50
BNP,^d^ ng/L	39 (23-65)	1.006 (1.003-1.009)	**<0.001**	1.007 (1.005-1.01)	**<0.001**
BNP *≥*127ng/L	38	10.2 (4.6-22.3)	**<0.001**	4.6 (1.8-11.5)	**0.001**
RVEDVi, mL/m^2^	114 (97-141)	1.01 (1.00-1.01)	0.05	1.01 (1.006-1.02)	**<0.001**
RVESVi, mL/m^2^	54 (42-70)	1.01 (1.006-1.03)	**0.001**	1.02 (1.01-1.03)	**<0.001**
RV EF, %	54 (47-59)	0.92 (0.80-0.96)	**<0.001**	0.90 (0.80-0.95)	**<0.001**
RV EF ≤47%	141	3.6 (1.7-7.8)	**0.001**	3.9 (1.9-8.2)	**<0.001**
RV EF ≤35 %	16	5.7 (2.2-15.3)	**<0.001**	6.4 (2.4-16.8)	**<0.001**
RV mass/volume, g/mL/m^2^	0.41 (0.36-0.48)	0.46 (0.02-11.30)	0.60	1.5 (0.06-36.80)	0.80
RVOT akinetic length, mm	34 (24-44)	1.04 (1.01-1.07)	**0.003**	1.05 (1.03-1.07)	**<0.001**
RVOT akinetic length ≥55 mm	47	3.2 (1.4-7.7)	**0.008**	3.90 (1.8-9.0)	**<0.001**
RAAi, cm^2^/m^2^	12 (11-15)	1.30 (1.20-1.40)	**<0.001**	1.20 (1.07-1.30)	**0.001**
RAAi ≥16 cm^2^/m^2^	66	2.4 (0.95-5.90)	0.06	2.5 (1.09-6.00)	**0.03**
LVEDVi, mL/m^2^	80 (69-92)	1.02 (1.01-1.03)	**0.001**	1.010 (1.00-1.03)	0.05
LVESVi, mL/m^2^	31 (25-40)	1.03 (1.01-1.04)	**<0.001**	1.02 (1.008-1.03)	**0.002**
LV EF, %	61 (56-66)	0.94 (0.90-0.97)	**<0.001**	0.9 (0.90-0.96)	**<0.001**
LV EF ≤55%	129	3.0 (1.4-6.6)	**0.004**	2.7 (1.3-5.8)	**0.008**
LV EF ≤35%	6	8.7 (2.6-28.9)	**<0.001**	7.8 (1.8-32.9)	**0.005**
RVLGE score	5(3-7)	1.5 (1.4-1.7)	**<0.001**	1.4 (1.3-1.6)	**<0.001**
RVLGE score ≥median	322	12.4 (2.9-52.8)	**0.001**	8.0 (2.4-26.7)	**0.001**
RVLGE score ≥upper quartile	121	22 (7.5-64.0)	**<0.001**	10.5 (4.6-23.7)	**<0.001**
LVLGE presence	41	7.2 (1.7-10.7)	**0.002**	5.9 (2.6-13.7)	**<0.001**
Pulmonary regurgitation, %	22 (4-36)	0.98 (0.96-1.01)	0.20	0.99 (0.98-1.02)	0.90
Restrictive RV physiology^e^	118 (26)	1.01 (0.30-3.10)	0.90	0.7 (0.20-2.20)	0.60
Restrictive RV physiology + RVEDVi ≥ 150 mL/m^2^^e^	16 (14)	2.7 (0.60-11.60)	0.20	3.5 (1.04-11.40)	**0.04**
Restrictive RV physiology + RVEDVi ≤115 mL/m^2^^e^	55 (47)	1.1 (0.3-3.8)	0.80	1.9 (0.7-5.7)	0.20
Tricuspid regurgitation ≥moderate	51 (9)	0.6 (0.1-2.5)	0.50	1.2 (0.4-3.6)	0.60
RVSP, mm Hg	37 (30-47)	0.99 (0.97-1.02)	0.80	1.02 (1.004-1.040)	**0.01**
RVSP *≥*47 mm Hg	113 (21)	1.4 (0.6-3.2)	0.40	2.5 (1.2-5.3)	**0.01**
TAPSE, mm	15 (12-18)	0.95 (0.86-1.04)	0.30	0.9 (0.80-1.04)	0.30
LV E/A ratio	1.6(1.3-2)	1.5 (0.93-2.50)	0.09	1.3 (0.70-2.10)	0.30
LV E/E’ lateral wall	6.7 (5.3-8.5)	1.1 (1.0-1.3)	0.05	1.1 (0.9-1.2)	0.10
PVO_2_, mL/kg/min^f^	26.3 (21-31.3)	0.88 (0.81-0.94)	**<0.001**	0.93 (0.80-0.99)	**0.02**
PVO_2_ ≤17 mL/kg/m^2^	50	3.9 (1.8-8.8)	**0.001**	4.5 (1.5-7.9)	**0.003**
Inducible VT at PES	24/70 (34)	2.9 (0.5-16.5)	0.20	1.9 (0.4-7.9)	0.30
Nonsustained VT^g^	67/550 (12)	1.1 (0.4-3.0)	0.90	2.0 (0.8-4.7)	0.10
Sustained atrial arrhythmia	62/550 (11)	6.8 (3.2-14.6)	**<0.001**	2.9 (1.3-6.7)	**0.009**

Values are median (IQR), n (%), or n, unless otherwise indicated. Selected cutoffs for categorical variables were based on the top decile for BNP and RVOT akinetic length, top quartile for RVSP and lowest quartile for RVEF and LVEF, lowest decile for RVEF, LVEF, and PVO_2_.

EDVi = end-diastolic volume indexed to body surface; EF = ejection fraction; ESVi = end-systolic volume indexed to body surface area; LGE = late gadolinium enhancement; NYHA = New York Heart Association classification; PES = programmed electrophysiological study; RAAi = right atrial area indexed to body surface area; RV = right ventricle; LV = left ventricle; RVOT = right ventricular outflow tract; VA = ventricular arrhythmia; VT = ventricular tachycardia.

a Repair for tetralogy of Fallot with pulmonary atresia and no systemic-pulmonary collaterals in 44 (8%), double outlet RV variant in 13 (3%), with absent pulmonary valve in 10 (2%).

b Redo surgical pulmonary valve implantation occurred in 113 at baseline and 118 during follow-up. Percutaneous pulmonary valve implantation occurred in 32 during follow-up).

c 12-lead electrocardiogram was available in 500 (91%).

d B-type natriuretic peptide (BNP) was available in 384 (70%).

e Data on restrictive RV physiology were available in 447 (81%), Restrictive RV physiology + top decile RVEDVi (≥150 mL/m^2^) in 16 (14%) and lowest quartile RVEDVi (≤115 mL/m^2^) in 55 (47%). Right ventricular systolic pressure (RVSP) was available in 446 (81%), tricuspid annular planar excursion (TAPSE) in 473 (86%), LV E/A ratio in 509 (93%), and LV E/E’ in 477 (87%).

f Peak oxygen uptake (PVO_2_) was available in 423 (77%).

g Holter monitoring was available in 142 (26%). Nonsustained VT was recorded in 66 patients during follow-up (median 12 beats: [8-18], median total cycle length 350 ms: [300-3884 ms]). The *P* values in **bold** are statistically significant.

### All-cause mortality

**Figure 2:**
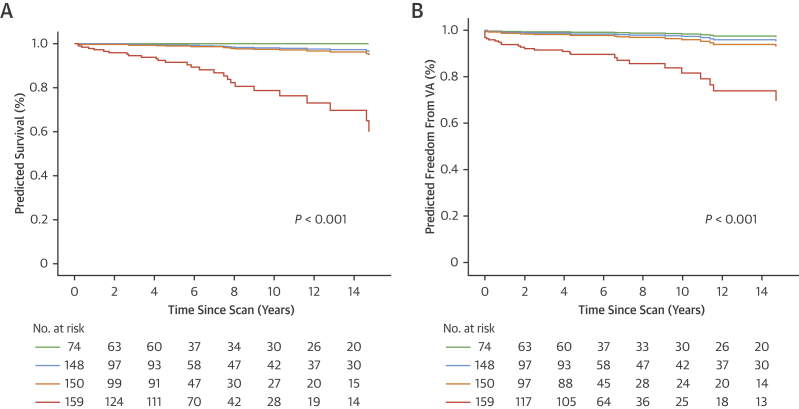
RV LGE Extent Predicts Mortality and VA Cox proportional hazard survival plots of RV LGE quartiles to predict VA **(A)** and all-cause mortality **(B)**. VA = ventricular arrythmia; other abbreviations as in Figure 1.

During the follow-up period, a total of 27 deaths were recorded (13 SCDs, 12 deaths caused by heart failure, and 2 noncardiac deaths). Univariable predictors are summarized in Table 1 and were consistent with previous reports/consensus aside our novel finding that supramedian RVLGE score (≥5) is associated with higher mortality. Mortality is related to RVLGE extent (Figure 2). NSVT, previous palliative shunt, ventriculotomy, and QRS duration >180 ms were not univariable predictors of mortality. Restrictive RV physiology was not predictive of mortality. In bivariable analysis, supramedian RVLGE remained an independent predictor of mortality (HR: 11.4; 95% CI: 2.7-48.8; *P =* 0.001), as did the presence of LVLGE, RV ejection fraction (EF) ≤35%, RVEF ≤47%, LVEF ≤55%, LVEF ≤35%, BNP levels ≥127 ng/L, PVO_2_ ≤17 mL/kg per minute, sustained atrial arrhythmia, and age ≥50 years (Supplemental Table 2).

### Risk score for predicting mortality

**Figure 3:**
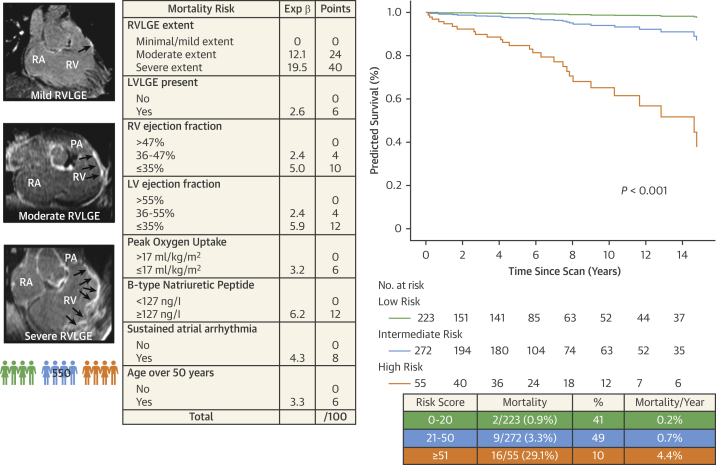
Annualized All-Cause Mortality Rate According to Risk Category Risk score with weighted independent predictors of mortality. Cox proportional hazard survival plot showing percentage survival for each risk category. Corresponding risk categories, mortality rate, and annualized mortality rate.

A total weighted-risk score ≥51 demonstrated a 93% specificity vs sensitivity 51% for predicting all-cause mortality and was chosen as the lower threshold for the highest risk of death category. Conversely a total score ≤20 had a sensitivity 93% vs specificity 42% and was used as the upper threshold for the low-risk category. The applied risk score (Figure 3) was a good discriminator of all-cause death (area under the curve [AUC]: 0.87; 95% CI: 0.78-0.95; *P <* 0.001). For every 1-point increase in risk score, there was an associated 7% increased risk of death (HR: 1.07; 95% CI: 1.05-1.08; *P <* 0.001). Freedom from this outcome at 3, 5, and 10 years was 89%, 87%, and 64%, respectively, for the high-risk category; 99%, 97%. and 94% for the intermediate-risk category; and 99% up to 10 years for the low-risk category.

This score performed better in predicting mortality when compared with other previously proposed risk models (8,11,18) (AUC: 0.87; 95% CI: 0.78-0.95; *P <* 0.001) (Table 2).

**Table 2:** Comparative Analysis of Performance Against Existing Risk Scores

Risk Model Applied for Prediction of Mortality	AUC ROC; *P* Value (CI)	
Babu-Narayan 2020	0.87; *P* < 0.001	(0.78-0.95)
Babu-Narayan without LGE 2020^a^	0.81; *P* < 0.001	(0.71-0.91)
Valente RVEF model 2014^b^	0.64; *P =* 0.02	(0.5-0.77)
Valente LVEF model 2014^c^	0.63; *P =* 0.03	(0.49-0.76)
Bokma 2017^d^	0.64; *P =* 0.01	(0.54-0.75)
Khairy without invasive data 2008^e^	0.56; *P =* 0.3	(0.46-0.65)

AUC = area under the curve; ROC = receiver-operating characteristic; other abbreviations as in Table 1.

a Scores were calculated using the model in Figure 3 without the inclusion of LGE cardiovascular magnetic resonance, given that LGE is not in routine clinical practice for this condition.

b To enable testing of existing models on our study cohort, points were allocated, using a similar approach to our study to the predictive cutoffs reported by Valente et al (8): 3 points = RV mass/volume ≥0.3 g/mL, history of atrial arrythmia, RVEF <48% in male/<50% in female individuals. 2 points = RV mass/volume ≥0.3 g/mL, RVEF <48% in male/<50% in female individuals. 1 point = RV mass/volume ≥0.3 g/mL.

c Points were allocated to the predictive cutoffs (8) as follows: 3 points = RV mass/volume ≥0.3 g/mL, history of atrial arrythmia, LVEF <55% in male/<54% in female individuals. 2 points = RV mass/volume ≥0.3 g/mL, LVEF <55% in male/<54% in female individuals. 1 point = RV mass/volume ≥0.3 g/mL.

d Patients were scored using the point allocation prescribed by Bokma et al (18).

e Calculated using the noninvasive parameters only given lack of invasive data available for most patients (11).

### Secondary analysis for VA and its prediction

**Figure 4:**
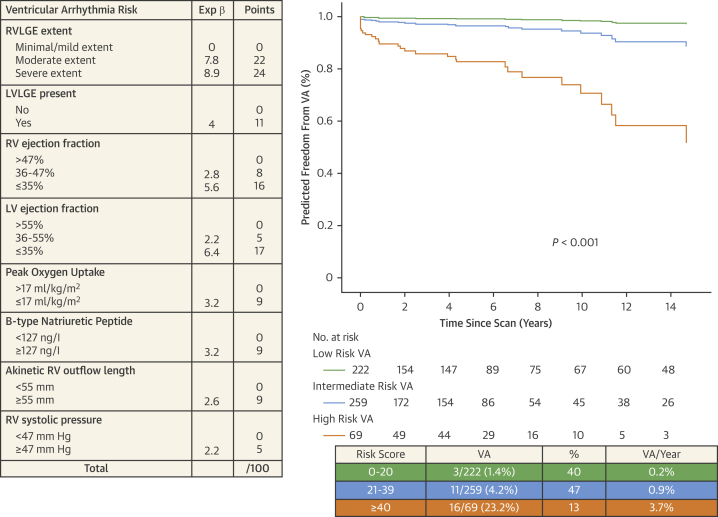
Annualized Rate of VA According to Risk Category Risk score with weighted independent predictors of VA. Cox proportional hazard survival plot showing freedom from VA for each category. Corresponding risk categories, mortality rate, and annualized mortality rate. PA = pulmonary artery; RA = right atrium; RV = right ventricle; other abbreviations as in Figures 1 and 2.

A total of 29 patients reached the VA composite endpoint (10 SCDs, 3 resuscitated VF events; 2 of whom had appropriate shock for VF, 1 resuscitated VF arrest and 16 with documented sustained VT). Freedom from VA was compromised as RVLGE extent increases (Figure 2). Univariable predictors are summarized in Table 1. Restrictive RV physiology was predictive of VA only when associated with RV dilation but was not independent of RV dilation alone. In bivariable analyses, RVLGE score ≥5 remained an independent predictor, as did LVLGE, RVEF ≤35%, RVEF ≤47%, LVEF≤55%, LVEF≤35%, PVO_2_ ≤17 mL/kg per minute, BNP levels ≥127ng/L, RVOT akinetic length ≥55 mm, and RVS*p ≥*47 mm Hg (Supplemental Table 2). A total weighted-risk score ≥40 demonstrated the most favorable specificity 91% vs sensitivity 52% for predicting VA, hence was chosen as the lower threshold for the highest risk of VA category. Conversely, a total score ≤20 with the most favorable sensitivity 90% over specificity 42% was used as the upper threshold for the low-risk category. The applied risk score (Figure 4) was also a good discriminator of the VA composite endpoint (AUC: 0.79; 95% CI: 0.69-0.88; *P <* 0.001). A 1-point increase in risk score was associated with a 7% increased chance of reaching the VA composite outcome (HR: 1.07; 95% CI: 1.05-1.09; *P <* 0.001). Freedom from this outcome at 3, 5, and 10 years for patients in the high-risk category was 81%, 79%, and 76%, respectively, compared with 98%, 97%, and 93% in the intermediate-risk and 99%, 99%, and 97% in the low-risk category.

### Histological assessment

**Figure 5:**
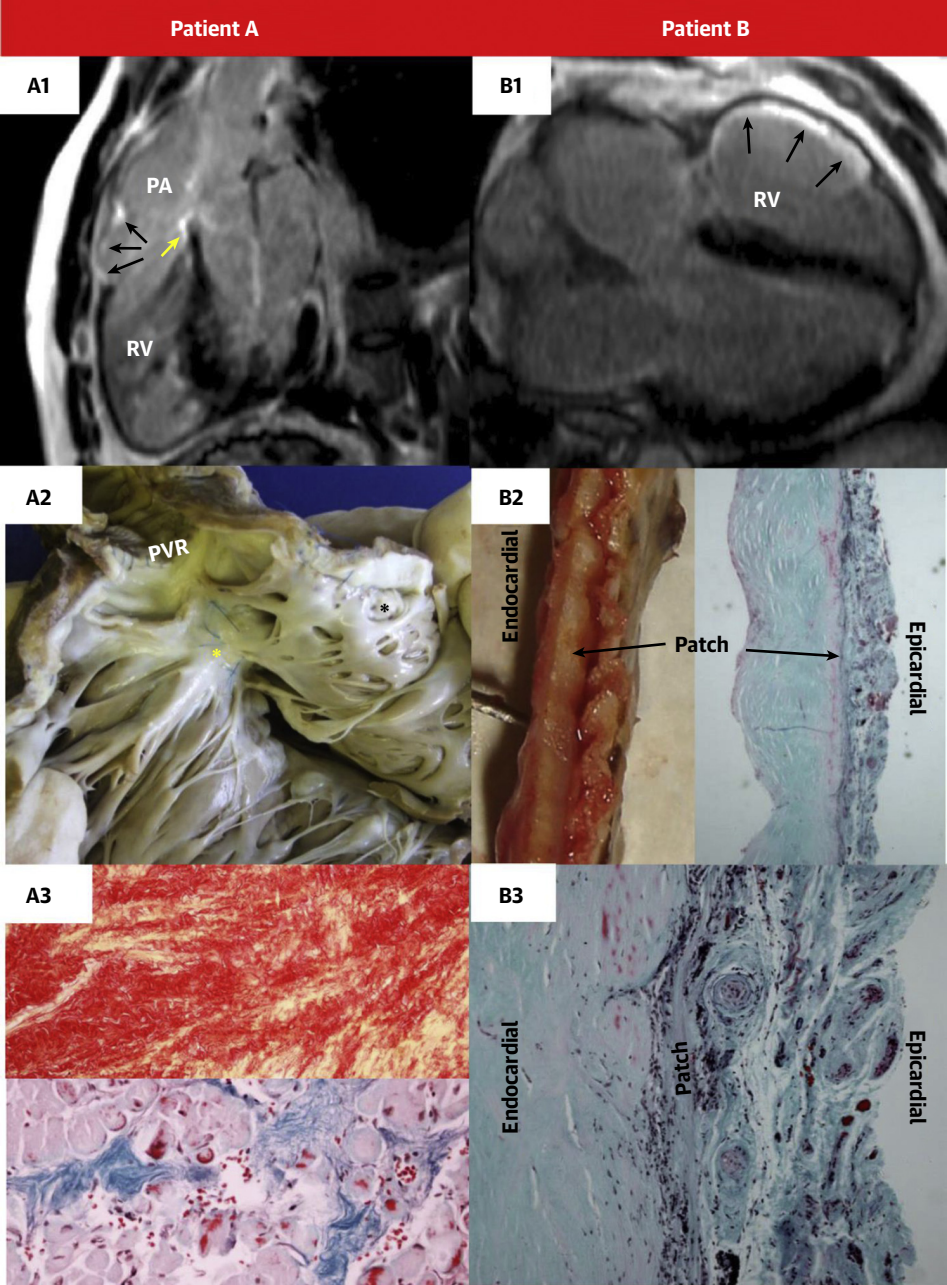
Correspondence Between Histological Fibrosis in an Explanted Heart and the Previous In Vivo LGE CMR Patient A **(left column)**: In vivo CMR **(A1)** showing LGE in the VSD patch site (**yellow arrow**) and RVOT **(black arrows)** below the PA. Postmortem macroscopic section of RV opened longitudinally **(A2)**. VSD patch site **(yellow asterisk)** and RVOT **(black asterisk)**. Microscopic examination (magnification ×200) of the RVOT **(A3)** confirmed the presence of extensive collagen (with Picrosirius Red stain, the collagen stained red and areas with myocardium stained yellow; magnification ×100). At higher magnification **×**200, with Masson’s Trichrome stain showing areas of collagen staining blue and myocardium pale red below. Patient B **(right column)**: LGE CMR in a patient with a childhood RVOT patch repair **(B1)** and RVOT LGE **(black arrows)**. Subsequent RVOT patch surgical excision at time of elective pulmonary valve replacement confirmed macroscopic **(B2 left)** and microscopic (**B2 right**; magnification ×16) fibrosis (**blue regions** on the Masson’s Trichome stain) with endothelialization over the epicardial and endocardial surface of the patch seen at higher magnification (×100) in **B3**. CMR = cardiovascular magnetic resonance; RVOT = right ventricular outflow tract; VSD = ventricular septal defect; other abbreviations as in Figures 1, 2, and 4.

In 1 patient who had sustained VT followed by SCD, there was visual correspondence between histological fibrosis in the explanted heart and the previous *in vivo* LGE CMR. In another patient, the RVOT patch was excised at the time of elective PVR, showing correlation, and the LGE CMR correlated with histological fibrosis over the epicardial surface of the patch (Figure 5).

## Discussion

We have shown how to identify patients with rTOF who are at high annual risk of death by using a weighted-risk score that integrates clinical, LGE CMR, exercise, and BNP measurement (Central Illustration). This performs better than previously proposed risk models (8,11,18). We have also enabled personalized risk stratification specific to malignant VA. This is the largest prospective study to date that also examines LGE extent and long-term outcomes, in a highly characterized adult rTOF cohort with a considerable follow-up period and hard clinical endpoints. We show for the first time that the extent of LGE is a significant and independent predictor of mortality, justifying its routine and periodic inclusion in the clinical surveillance of adults with rTOF.

**Central Illustration:**
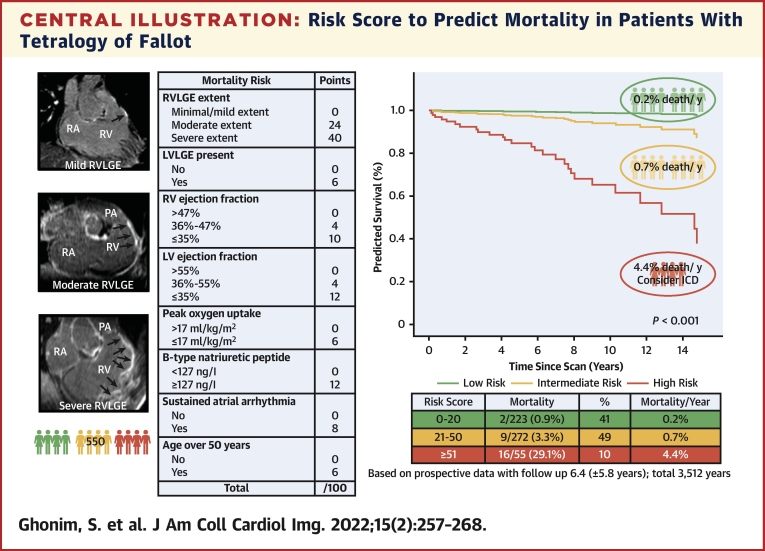
Risk Score to Predict Mortality in Patients With Tetralogy of Fallot Risk score identifies contemporary adult patients with repaired tetralogy of Fallot at high annual risk of death. Abbreviations as in Figures 1, 2, and 4.

### Mortality prediction in contemporary adults with rTOF

It is well-recognized that no single risk factor accurately predicts adverse outcome in patients with rTOF. A 2008 multifactorial risk score from a retrospective multicenter study (9) was pioneering. However, the previous study included patients who were already considered high risk with significant PR and included recurrent events in those with secondary prevention ICDs, hence limiting its application. Furthermore, invasive tests (LV end-diastolic pressure and VT inducibility) included in this 2008 risk score are not considered pragmatic in unselected patients, especially for serial study. The addition of LGE CMR to the risk assessment armamentarium that we propose here is noninvasive, hence safer and more applicable. In addition, other noninvasive risk predictors for outcomes have since been described (8,9,15,17). In keeping with the largest multicenter observational registry study to date (International Multicenter TOF Registry), atrial arrhythmia and CMR-derived RVEF and LVEF were predictors of outcome in our study (8). Prospective studies have also identified other CMR-derived factors including RAAi ≥16 cm^9^ and increased RVOT akinetic length (9), reduced peak VO_2_, (15,16) increased BNP, (17) and increased RVSP (8). These parameters were also univariable predictors of outcome in our study. In contrast, QRS duration >180 ms, previous palliative shunt, or ventriculotomy were not predictive of outcome. This may relate to the changing profiles of more contemporary adult rTOF cohorts over the past few decades (1,19,20). In the recent era, a more conservative approach to RVOT reconstruction, avoidance of ventriculotomy with a trans-atrial/trans-pulmonary approach, and primary repair at a much younger age has evolved. Our study population reflects our tertiary center’s practice of being proactive in treating asymptomatic patients with PR before RV dysfunction ensues as per evolving consensus criteria (4,21). Hence we cannot infer that the lack of association between PR and outcomes implies PR is not a risk factor.

### Prediction of life-threatening VA for guiding primary prevention ICD indication

Clinicians and patients want more clarity and precision for the selection of patients with rTOF for primary prevention ICD. In acquired heart disease, a survival benefit from primary prevention ICD was demonstrated in patients with a minimum 3.5% annual risk of SCD (22). In our study, we have identified addressable high-risk groups of patients that have an estimated 4.4% annual risk of mortality and 3.7% risk of VA who could be considered for primary prevention with ICD or VT ablation. These patients comprise 10% and 13% of the total cohort of patients with rTOF, respectively. On the other end of the risk spectrum, patients in the low-risk category had only 0.2% annual risk of VA, thus can be reassured. We anticipate that a reevaluation of risk would be triggered with change in clinical status or when a routinely timed noninvasive test shows change or after a structural intervention; LGE would not be added to CMR study at every visit. We, like others (1,23), found NSVT to be benign; it was neither associated with mortality nor significant VA, calling into question guidelines suggesting NSVT should be considered for ICD or its use as a surrogate secondary endpoint in rTOF. Our secondary composite endpoint for VA did not include appropriate ICD shock to ensure it was robust and avoid concerns that appropriate ICD (24) therapy could be delivered for potentially benign NSVT. Furthermore, our study was to predict prognosis and not device outcomes (11).

### Study Limitations

This was a single-center study, yet our cohort was large and followed for a long period, with rTOF repair at many centers (7 in the United Kingdom and other international), hence reflective of various surgical eras and approaches and representative of this heterogeneous population. RVLGE CMR acquisition requires training to avoid false negative reporting (14), although recent CMR sequences have made LGE acquisition less operator-dependent, making wider uptake easier (25) and in future enabling comprehensive high-resolution coverage (26,27). We continued to use our previously published RV segmental scoring system for LGE (14), given its high reproducibility and its simplicity, and for consistency in this prospective study. Signal-intensity–based thresholds might be considered an alternative for quantifying RVLGE but are limited by partial volume effects, sternal wire artifact, epicardial fat, and the thin RV wall. No studies to date have validated this in the uniquely shaped RV after rTOF repair (28,29).

External validation of these risk score algorithms in a new cohort will be possible once similar data are collected systematically at scale.

### Future direction

RVLGE was heavily weighted in both risk scores caused by its strong relative prognostic value. Future studies of total fibrosis burden will also quantify LV interstitial fibrosis (T1 mapping CMR) and require bespoke approaches for the RV (30) and there may be ways to measure fibrosis activity. Machine learning could help timely incorporation of newly discovered risk factors, including molecular signatures of fibrosis or other relevant measures, allowing even more personalized clinical care.

## Conclusions

Most of the growing population of adults living with rTOF can expect long and healthy lives, but a small minority are at much higher risk for premature cardiovascular death. For the first time, we show LGE extent is prognostic, justifying its inclusion in clinical practice. We present a weighted-risk score to identify the subgroup of patients with rTOF who are at high annual risk of death who may benefit from targeted therapy with ICDs, VT ablation, or heart failure therapy.

Perspectives **COMPETENCY IN PATIENT CARE AND PROCEDURAL SKILLS:** Current risk stratification for premature death among patients with rTOF is inadequate, and there is no robust and easily applicable system for clinicians to use to leverage multiple risk factors objectively when treating individual patients. This study adds a clinically applicable integrated risk score, devised by weighting appropriately all independent predictors of mortality, including clinical, CMR, exercise, and BNP measures to identify the subgroup of high-risk patients with rTOF who have a more than 4% chance of dying per year. LGE extent was shown for the first time to be a strong predictor of mortality in patients with rTOF. The risk score is a step toward better selection of high-risk patients with rTOF who may benefit from targeted therapy.

**TRANSLATIONAL OUTLOOK:** The proposed risk identifies patients at high risk of annualized death. LGE CMR is justified in the lifelong surveillance of patients with rTOF.

## Funding Support and Author Disclosures

This work was supported by the British Heart Foundation (FS/11/38/28864), Drs Babu-Narayan and Heng were funded by the British Heart Foundation. Prof Dudley Pennell is a consultant to Siemens. All other authors have reported that they have no relationships relevant to the contents of this paper to disclose.

## References

[1] Gatzoulis M.A., Balaji S., Webber S.A. (2000). Risk factors for arrhythmia and sudden cardiac death late after repair of tetralogy of Fallot: a multicentre study. Lancet.

[2] Nollert G., Fischlein T., Bouterwek S. (1997). Long-term survival in patients with repair of tetralogy of Fallot: 36-year follow-up of 490 survivors of the first year after surgical repair. J Am Coll Cardiol.

[3] Silka M.J., Hardy B.G., Menashe V.D. (1998). A population-based prospective evaluation of risk of sudden cardiac death after operation for common congenital heart defects. J Am Coll Cardiol.

[4] Baumgartner H., De Backer J., Babu-Narayan S.V. (2021). 2020 ESC Guidelines for the management of adult congenital heart disease. The Task Force for the management of adult congenital heart disease of the European Society of Cardiology (ESC). Eur Heart J.

[5] Stout K.K., Daniels C.J., Aboulhosn J.A. (2019). AHA/ACC guideline for the management of adults with congenital heart disease: a report of the American College of Cardiology/American Heart Association Task Force on Clinical Practice Guidelines. J Am Coll Cardiol.

[6] Geva T., Mulder B., Gauvreau K. (2018). Preoperative predictors of death and sustained ventricular tachycardia after pulmonary valve replacement in patients with repaired tetralogy of Fallot enrolled in the indicator cohort. Circulation.

[7] Bokma J.P., Geva T., Sleeper L.A. (2018). A propensity score-adjusted analysis of clinical outcomes after pulmonary valve replacement in tetralogy of Fallot. Heart.

[8] Valente A.M., Gauvreau K., Assenza G.E. (2014). Contemporary predictors of death and sustained ventricular tachycardia in patients with repaired tetralogy of Fallot enrolled in the INDICATOR cohort. Heart.

[9] Bonello B., Kempny A., Uebing A. (2013). Right atrial area and right ventricular outflow tract akinetic length predict sustained tachyarrhythmia in repaired tetralogy of Fallot. Int J Cardiol.

[10] Diller G.P., Kempny A., Liodakis E. (2012). Left ventricular longitudinal function predicts life-threatening ventricular arrhythmia and death in adults with repaired tetralogy of Fallot. Circulation.

[11] Khairy P., Harris L., Landzberg M.J. (2008). Implantable cardioverter-defibrillators in tetralogy of Fallot. Circulation.

[12] Bedair R., Babu-Narayan S.V., Dimopoulos K. (2015). Acceptance and psychological impact of implantable defibrillators amongst adults with congenital heart disease. Int J Cardiol.

[13] Yap S.C., Roos-Hesselink J.W., Hoendermis E.S. (2006). Outcome of implantable cardioverter defibrillator s in adults with congenital heart disease: a multi-centre study. Eur Heart J.

[14] Babu-Narayan S.V., Kilner P.J., Li W. (2006). Ventricular fibrosis suggested by cardiovascular magnetic resonance in adults with repaired tetralogy of Fallot and its relationship to adverse markers of clinical outcome. Circulation.

[15] Giardini A., Specchia S., Tacy T.A. (2007). Usefulness of cardiopulmonary exercise to predict long-term prognosis in adults with repaired tetralogy of Fallot. Am J Cardiol.

[16] Babu-Narayan S.V., Diller G.P., Gheta R.R. (2014). Clinical outcomes of surgical pulmonary valve replacement after repair of tetralogy of Fallot and potential prognostic value of preoperative cardiopulmonary exercise testing. Circulation.

[17] Heng E.L., Bolger A.P., Kempny A. (2015). Neurohormonal activation and its relation to outcomes late after repair of tetralogy of Fallot. Heart.

[18] Bokma J.P., de Wilde K.C., Vliegen H.W. (2017). Value of cardiovascular magnetic resonance imaging in noninvasive risk stratification in tetralogy of Fallot. JAMA Cardiol.

[19] Gatzoulis M.A., Till J.A., Somerville J., Redington A.N. (1995). Mechanoelectrical interaction in tetralogy of Fallot: QRS prolongation relates to right ventricular size and predicts malignant ventricular arrhythmias and sudden death. Circulation.

[20] Cuypers J.A., Menting M.E., Konings E.E. (2014). Unnatural history of tetralogy of Fallot: prospective follow-up of 40 years after surgical correction. Circulation.

[21] Heng E.L., Gatzoulis M.A., Uebing A. (2017). Immediate and midterm cardiac remodeling after surgical pulmonary valve replacement in adults with repaired tetralogy of Fallot: a prospective cardiovascular magnetic resonance and clinical study. Circulation.

[22] Bardy G.H., Lee K.L., Mark D.B. (2005). Amiodarone or an implantable cardioverter–defibrillator for congestive heart failure. N Engl J Med.

[23] Cullen S., Celermajer D.S., Franklin R.C. (1994). Prognostic significance of ventricular arrhythmia after repair of tetralogy of Fallot: a 12-year prospective study. J Am Coll Cardiol.

[24] Waldmann V., Bouzeman A., Duthoit G. (2020). Long-term follow-up of patients with tetralogy of Fallot and implantable cardioverter defibrillator: the DAI-T4F Nationwide Registry. Circulation.

[25] Keegan J., Jhooti P., Babu-Narayan S.V. (2014). Improved respiratory efficiency of 3D late gadolinium enhancement imaging using the continuously adaptive windowing strategy (CLAWS). Magn Reson Med.

[26] Kellman P., Larson A.C., Hsu L.Y. (2005). Motion-corrected free-breathing delayed enhancement imaging of myocardial infarction. Magn Reson Med.

[27] Ghonim S., Ernst S., Keegan J. (2020). Three-dimensional late gadolinium enhancement cardiovascular magnetic resonance predicts inducibility of ventricular tachycardia in adults with repaired tetralogy of Fallot. Circ Arrhythm Electrophysiol.

[28] Flett A.S., Hasleton J., Cook C. (2011). Evaluation of techniques for the quantification of myocardial scar of differing etiology using cardiac magnetic resonance. J Am Coll Cardiol Img.

[29] Rydman R., Gatzoulis M.A., Ho S.Y. (2015). Systemic right ventricular fibrosis detected by cardiovascular magnetic resonance is associated with clinical outcome, mainly new-onset atrial arrhythmia, in patients after atrial redirection surgery for transposition of the great arteries. Circ Cardiovasc Imaging.

[30] Broberg C.S., Huang J., Hogberg I. (2016). Diffuse LV myocardial fibrosis and its clinical associations in adults with repaired tetralogy of Fallot. J Am Coll Cardiol Img.

