# Supplementary material for: Predicting Survival in Repaired Tetralogy of Fallot: A Lesion-Specific and Personalized Approach
Source: JACC Cardiovasc Imaging. 2022 Feb;15(2):257–68. doi: 10.1016/j.jcmg.2021.07.026 (PMC8821017; doi:10.1016/j.jcmg.2021.07.026)
Supplement: Supplemental Tables 1 and 2 [file mmc1.doc]

**SUPPLEMENTAL MATERIAL**

| **Table S1.** Outcomes at study end at mean 6.4 years (±5.8 years) follow-up from index CMR | | | | |
| --- | --- | --- | --- | --- |
| **Outcomes at study end** | **All patients** | **Supra-median RVLGE extent** | **Infra-median RVLGE extent** | **P-value** |
| **All-cause mortality at study end ^*^** | **27** | **25** | **2** | **<0.001** |
| **Cardiovascular mortality** ^†^ | **25** | **24** | **1** | **<0.001** |
| **Ventricular arrhythmia - life threatening**^‡^ | **29** | **26** | **3** | **<0.001** |
| Clinical sustained ventricular tachycardia ^§^ | 16 | 15 | 1 | **0.006** |
| Sudden cardiac death | 13 | 12 | 1 | **0.02** |
| Heart failure death ^§^ | 12 | 12 | 0 | **0.002** |
| Resuscitated VF ^#^ | 3 | 3 | 0 | 0.3 |

*****All-cause mortality included 27 deaths (25 cardiovascular deaths and 2 non-cardiac deaths secondary to metastatic breast cancer and a head injury). † There were 25 cardiovascular deaths (13 sudden cardiac death; SCD and 12 heart failure deaths). ^‡^There were 29 ventricular arrhythmia (VA) composite outcome events (16 clinical sustained VT, 10 SCD and 3 resuscitated VF). ^§^Clinical sustained VT≥30 seconds duration or haemodynamically significant VT requiring cardioversion. ^§^Heart failure related deaths included 2 perioperative deaths caused by heart failure. ^#^Resuscitated ventricular fibrillation comprised 1 patient successfully resuscitated from cardiac arrest with ventricular fibrillation and 2 patients receiving appropriate shocks for ventricular fibrillation.

**Table S2**

|  | **All-cause mortality** | **P-value** | **Ventricular arrhythmia** | **P-value** |
| --- | --- | --- | --- | --- |
|  | **Hazard Ratio (95%** |  | **Hazard Ratio (95%** |  |
| RVLGE score ≥median | 10.8(1.05-6.5) | **0.001** | 6.6 (1.9-22) | **0.002** |
| LVLGE presence | 2.6 (1.04-6.5) | **0.03** | 4 (1.7-9.2) | **0.001** |
| RVLGE score ≥median | 10.4 (2.4-44) | **0.002** | 7.3(2.2-24.8) | **0.001** |
| Age ≥50 years | **3.3 (1.5-7.5)** | **0.004** | 2.3(0.97-5.6) | 0.06 |
| RVLGE score ≥median | 12.2(2.8-52) | **0.001** | 7.6 (2.3-25.7) | **0.001** |
| Age at repair ≥2 years | 1.2(0.4-3.5) | 0.7 | 1.7 (0.6-4.8) | 0.3 |
| RVLGE score ≥median | 10.7(2.5-46.2) | **0.001** | 7.4(2.2-25) | **0.001** |
| Palliative shunt | 1.8 (0.8-4) | 0.1 | 1.3 (0.6-2.6) | 0.5 |
| RVLGE score ≥median | 8.8 (2.04-38.6) | **0.004** | 7.1 (2.1-23.9) | **0.002** |
| BNP ≥127 ng/l | 6.2 (2.8-13.8) | **<0.001** | 3.2 (1.3-7.9) | **0.01** |
| RVLGE score ≥median | 9.9 (2.3-43.1) | **0.002** | 6.3(1.9-21.4) | **0.003** |
| RV EF ≤47% | 2.4 (1.1-5.3) | **0.02** | 2.8 (1.3-6) | **0.006** |
| RVLGE score ≥median | 12.1 (2.8-51.7) | **0.001** | 7.8 (2.3-26.1) | **0.001** |
| RV EF ≤35% | 5 (1.9-13.5) | **0.001** | 5.6 (2.1-14.9) | **<0.001** |
| RVLGE score ≥median | 11 (2.6-47.6) | **0.001** | 6.8 (2-23) | **0.002** |
| RVOT akinetic length ≥55mm | 2 (0.8-4.8) | 0.1 | 2.6 (1.1-5.9) | **0.02** |
| RVLGE score ≥median | 11.7 (2.7-50) | **0.001** | 7.5 (2.2-25) | **0.001** |
| RAA ≥16cm^2^/m^2^ | 1.6 (0.6-4.1) | 0.3 | 1.8 (0.7-4.4) | 0.2 |
| RVLGE score ≥median | 11.1 (2.5-47.5) | **0.001** | 7.3 (2.1-24.5) | **0.001** |
| LV EF ≤55% | 2.4 (1.1-5.2) | **0.02** | 2.2 (1.06-4.7) | **0.03** |
| RVLGE score ≥median | 11.4 (2.7-48.8) | **0.001** | 7.8 (2.3-26.2) | **0.001** |
| LV EF ≤35% | 5.9 (1.8-20) | **0.004** | 6.4 (1.5-27.3) | **0.01** |
| RVLGE score ≥median | 12.2(2.9-52.2) | **0.001** | 7.5(2.2-25) | **0.001** |
| RVSP ≥47mmHg | 1.1(0.5-2.6) | 0.8 | 2.2(1.07-4.7) | **0.03** |
| RVLGE score ≥median | 11.3 (2.6-48) | **0.001** | 7.7 (2.3-25.8) | **0.001** |
| PVO_2_ ≤17 ml/kg/m^2^ | 3.2 (1.5-7.4) | **0.004** | 3.2(1.4-7.4) | **0.005** |
| RVLGE score ≥median | 8.7 (2-37.7) | **0.004** | 7.1 (2.1-24) | **0.002** |
| Prior sustained atrial arrhythmia | 4.3 (2-9.3) | **<0.001** | 2 (0.9-4.5) | 0.1 |
| RVLGE score ≥median | **19.7 (4.6-84.6)** | **<0.001** | **6.8(2.5-18.1)** | **<0.001** |
| Restrictive RV physiology + RVEDVi ≥ 150ml/m^2^ | 1.5(0.3-6.4) | 0.6 | 2.7(2.5-18.1) | 0.1 |
| RVLGE score ≥median | 12.6(2.9-53.9) | **0.001** | 7.6(2.3-25.5) | **0.001** |
| Non-sustained VT | 0.8 (0.3-2.4) | 0.7 | 1.7(0.7-3.9) | 0.2 |

BNP; B-type natriuretic peptide, EDVi; end-diastolic volume indexed to body surface area, EF; ejection fraction,

ESVi; end-systolic volume indexed to body surface area, LGE; late gadolinium enhancement, LV; left ventricle, PVO_2_; peak oxygen uptake; RAAi; right atrial area indexed to body surface area, RV; right ventricle, RVOT; right ventricular outflow tract, RVSP; right ventricular systolic pressure
